# Heparin and Its Derivatives: Challenges and Advances in Therapeutic Biomolecules

**DOI:** 10.3390/ijms221910524

**Published:** 2021-09-29

**Authors:** Nipa Banik, Seong-Bin Yang, Tae-Bong Kang, Ji-Hong Lim, Jooho Park

**Affiliations:** 1 Department of Integrated Biosciences, Graduate School, BK21 Program, Konkuk University, Chungju 27478, Korea; baniknipa@gmail.com (N.B.); tjdqls414@gmail.com (S.-B.Y.); kangtbko@kku.ac.kr (T.-B.K.); jhlim@kku.ac.kr (J.-H.L.); 2 Department of Biomedical Chemistry, College of Biomedical & Health Science, Konkuk University, Chungju 27478, Korea

**Keywords:** heparin, heparin derivative, polysaccharide, anti-cancer effect, bioconjugate

## Abstract

Heparin has been extensively studied as a safe medicine and biomolecule over the past few decades. Heparin derivatives, including low-molecular-weight heparins (LMWH) and heparin pentasaccharide, are effective anticoagulants currently used in clinical settings. They have also been studied as functional biomolecules or biomaterials for various therapeutic uses to treat diseases. Heparin, which has a similar molecular structure to heparan sulfate, can be used as a remarkable biomedicine due to its uniquely high safety and biocompatibility. In particular, it has recently drawn attention for use in drug-delivery systems, biomaterial-based tissue engineering, nanoformulations, and new drug-development systems through molecular formulas. A variety of new heparin-based biomolecules and conjugates have been developed in recent years and are currently being evaluated for use in clinical applications. This article reviews heparin derivatives recently studied in the field of drug development for the treatment of various diseases.

## 1. Introduction

Heparan sulfate (HS) is a natural component of the extracellular matrix (ECM) that is abundantly expressed at cell surfaces in vertebrate tissues as part of proteoglycans (PGs). These proteoglycans carry branches of glycosaminoglycans (GAG) improving a lot of critical physiological processes. Sulfated natural GAG molecules are chondroitin sulfate (CS), heparin, heparan sulfate (HS), keratan sulfate, and dermatan sulfate (DS); non-sulfated natural GAG molecule is Hyaluronic acid (HA). Considering the disadvantages of the direct use of natural GAG, GAG-mimetic biomolecules have become the key of biofunctions these days [1]. Among GAGs, HS is composed of D-glucosamine and hexuronic acid units with many sulfate groups in a molecular structure and shows good biocompatibility and biodegradability [2]. It is a biomaterial that is essential for normal embryonic development, cellular homeostasis, and various pathological processes as well as neurodegenerative diseases [3,4,5]. For example, abnormal heparan sulfate storage in lysosomes develops primarily mucopolysaccharidosis lll B [6].

Heparin, whose structure and properties are similar to those of heparan sulfate, has been used clinically as an anticoagulant. Heparin is an FDA (Food and Drug Administration)-approved drug used for patients at the risk of blood clots. Heparin is found in animal tissues in forms such as heparan sulfate but differs in that it is used in medicines based on its strong anticoagulation effects [7]. Although heparin is mostly used as an anticoagulant, it has great potential for use as a biomolecule for the treatment of inflammation, injury, or malignant tumors [8]. In addition, the various advantages of heparin have led to the development of similar biomolecules that mimic it. For example, different types of heparin mimetics were developed as di- and tri-block copolymers containing anionic poly (sodium 2-acrylamido-2-methylpropane sulfonate (PAMPS) acting as an anticoagulant component with polyethylene glycol (PEG) [9].

Heparin and its derivatives have been clinically developed to optimize the anticoagulant effect while decreasing the systemic toxicity [10,11]. Heparin is a linear macromolecule consisting of a heterogeneous mixture of saccharide chains. Unfractionated heparin (UFH) is a naturally occurring glycosaminoglycan ranging in molecular weight (MW) from approximately 16,000 Da [12]. The problem with the use of UFH as a safe medicine is that its molecular size is not optimized for regulating the blood coagulation process or target inhibition. The average molecular weight of UFH is much greater than the small heparin binding site of antithrombin or Factor Xa (58,000 Da), which is a target for exerting anticoagulant effects. Low-molecular-weight heparins (LMWHs) derived from UFH have been widely used due to their excellent efficacy and low number of side effects due to their optimized molecular size. For many indications, LMWH is usually preferred over UFH due to its good predictability, low number of side effects, and lower risk of inducing bleeding [6]. In this line, smaller heparins such as very-low-molecular-weight heparins (VLMWHs) or ultra-low-molecular-weight heparins (ULMWHs) have also been introduced by researchers. Furthermore, a small synthetic heparin pentasaccharide named fondaparinux was developed as an alternative anticoagulant with an optimized molecular weight and sequence; relatively, a small heparin derivative with a small molecular weight is easy to synthesize.

**Figure 1:**
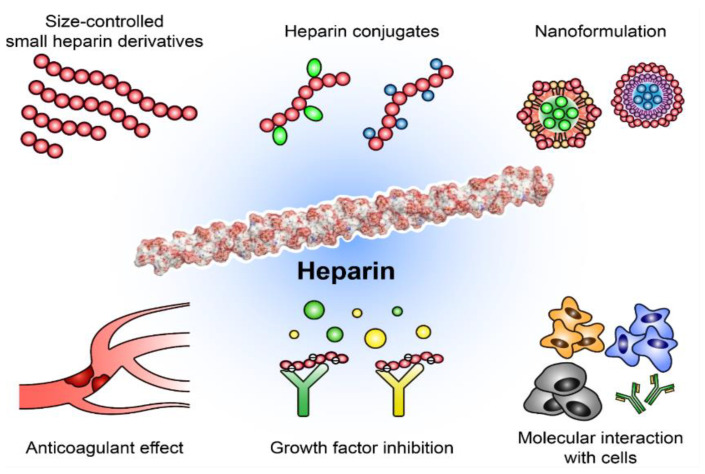
Schematic illustration of heparin derivatives and applications. Various derivatives have increased the variety of therapeutic uses available, which can serve not only as anticoagulants, but also as functional biomolecules for diverse diseases.

Studies of various medical uses of heparin derivatives are not limited to analyses of their anticoagulative effects. To date, many heparin derivatives and conjugates have been developed as new drug candidates or theranostic (diagnosis and therapy) agents like probes. Their therapeutic effects have been studied for use in anti-cancer, wound-healing, anti-viral, and anti-inflammatory therapies [13,14]. In particular, a range of studies suggest that heparin derivatives and conjugates can inhibit tumor growth and metastasis by suppressing many tumor-related factors (Figure 1) [1,10,11]. Heparin-based biomolecules are able to bind to vascular endothelial growth factor (VEGF), basic fibroblast growth factor (bFGF), P-selectin, CXC motif chemokine ligand 12 (CXCL12, also called stromal-derived factor-1; SDF-1), and heparanase, affecting cell migration, adhesion, and angiogenesis [15,16,17]. Therefore, in this review, we summarize the current advances and challenges in the development of heparin-related biomolecules such as heparin derivatives and conjugates for their successful clinical application.

## 2. Limitations and Challenges for Using Heparins for Medical Purposes

The decades-long clinical use of heparin and heparin derivatives has recently been challenged due to the development of competitive drugs. Heparin is an FDA-approved safe drug, but other competitive anticoagulants that can be used orally have been emerging in the pharmaceutical market. It has been well documented that the administration of UFH or LMWH is the current safe and standard treatment for deep vein thrombosis (DVT), pulmonary embolism, and stroke prevention. The safety and efficacy of the use of heparin have been confirmed over decades-long studies, and heparin is still in clinical use in many high-risk patients [18,19]. However, UFH and LMWHs are difficult for the body to absorb when administered orally because they have large molecular weights of over 4000 Da and exhibit strong negative surface charges [20,21]. Since even very small molecular heparins exceed 1500 Da, normal heparin derivatives also cannot be absorbed in the gastrointestinal (GI) tract when they are administered orally [22]. However, in the case of newly developed direct oral anticoagulants (DOACs), which act on similar targets with low molecular weights, they are able to be used as oral medicine for patients [23,24]. For this reason, DOACs including rivaroxaban and apixaban are increasingly being used to prevent venous thromboembolism after surgery or arthroplasty [25]. Considering the importance of anticoagulants, which should be used daily for preventive purposes, DOACs may continue to increase their share of the anticoagulant market as a substitute for heparins.

With the various studies of heparin as functional biomolecules, heparin and its derivatives have been found to have various therapeutic functions in treating disease. In particular, the molecular structure of heparin is similar to that of HS, a component of the ECM, and is capable of biological interactions with various proteins and cytokines [3,5]. For example, the currently used heparin derivatives could impact cancer progression in cancer patients via anti-angiogenic and anti-metastatic effects [26]. The administration of LMWH to cancer patients increased the survival rate in a manner that was not related to the anticoagulant effect of heparin [27,28,29]. Additionally, heparins could act as effective wound-healing accelerators, increasing angiogenesis; as anti-viral agents that can inhibit human immunodeficiency virus; and as regulators of inflammatory arthritis, inhibiting cell accumulation and collagen destruction [11,30,31]. In addition, several heparin derivatives have been developed for non-anticoagulant applications. However, in order to use a heparin derivative for therapeutic purposes other than its anticoagulant effect, its anticoagulant effect needs to be eliminated. Additional molecular modifications such as periodate treatment, molecular modification, and size control can be used to eliminate the anticoagulant effect, but these methods have not been completely validated in clinical studies [32]. It seems that the chemical conjugation of heparins with other molecules could result in an increase in biological activity or therapeutic effect with the loss of the intrinsic anticoagulant properties of heparin. Thus, an optimized heparin conjugate might be a good drug candidate. Therefore, it is important to develop and study an appropriate heparin derivative or conjugate suitable for potential clinical use.

## 3. Various Heparin Derivatives and Conjugates for Optimizing Anticoagulant Effect

### 3.1. Heparin Conjugates for Optimizing Anticoagulant Effects

**Figure 2:**
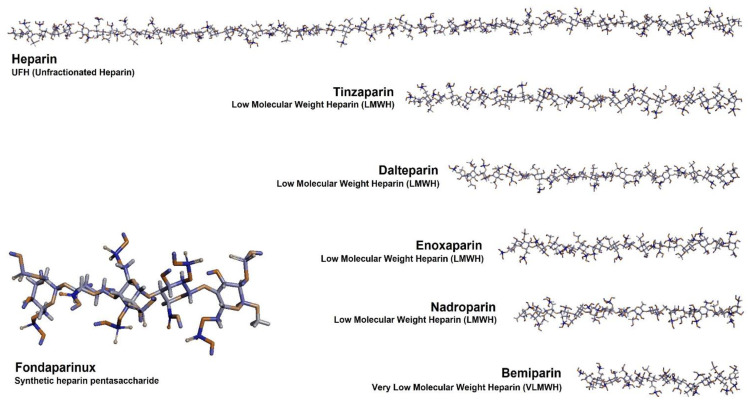
Chemical structures of unfractionated heparin (UFH), low-molecular-weight heparin (LMWH), very-low-molecular-weight heparin (VLMWH), and synthetic heparin pentasaccharide used as anticoagulants. Based on the main sequence of heparin, the molecular structure of heparin was specified through computer simulation to consider the molecular interaction with heparin related proteins. The molecules were generated from the solution structures of heparin obtained using an X-ray scattering model (protein data bank; PDB, 3IRL) [38]. The molecular structures of heparin were further modified using the ChemDraw 20.1 Professional (PerkinElmer) program based on their synthetic processes and average molecular sizes. The modified molecules were visualized using the Discovery Studio 2021 (BIOVIA) software.

To overcome several problems of UFH, such as unwanted interactions with plasma proteins, low-molecular-weight heparin derivatives have been prepared and clinically used (Figure 2). The optimal heparin molecular structure and size might be derived through structural analysis of heparin based on computer simulation. Smaller heparins such as LMWH have shown better pharmacokinetic and pharmacodynamic profiles because their molecular size is optimized for targeting Factor Xa to exert anticoagulative effects [33,34]. Commercially available LMWHs include enoxaparin, nadroparin, tinzaparin, reviparin, and dalteparin [35]. Although their anticoagulant effects are more predictable and dose-dependent than those of UFHs, they have higher anti-Xa/anti-lla activity ratios compared with UFH. Among the LMWHs, tinzaparin and dalteparin have molecular weights that are higher than those of others by 1000 to 2000 Da [12]. On the other hand, the average molecular weights of enoxaparin and nadroparin are 4300–4500 Da. LMWH, those who have low molecular weight, have low activity against Factor lla (thrombin). Therefore, LMWH derivatives have a higher ratio of anti-Xa activity to anti-lla activity [36]. These substances are widely used across the world as anticoagulants [7,37]. There are subtle differences in the structure of the two LMWHs. Enoxaparin, for instance, is usually prepared from UFH via a chemical b-elimination reaction so that 1,6-anhydrosugar residues are present at the reducing end. However, nadroparin does not have a reducing end because it is obtained by nitrous acid depolymerization.

It has been reported that very-low-molecular-weight heparins or synthetic low molecular heparin mimics can show Factor Xa-specific anticoagulant effects, resulting in a decrease in systemic toxicity [39,40,41]. The heparin binding site of antithrombin can accommodate a small number (five or more) of polysaccharide residues at high affinity sequence. In fact, the number of heparin saccharide rings that can bind effectively to antithrombin is limited to approximately five, meaning that very small heparins with an average molecular weight of 1800–2500 Da are sufficient for molecular binding [33,42]. Based on the structural information of the small heparin binding site, VLMWHs including semuloparin and bemiparin have been prepared and studied [43,44]. Furthermore, a size-optimized synthetic heparin mimic was chemically synthesized and developed, exploiting the appropriate heparin size and sequences [45]. Fondaparinux, a synthetic heparin pentasaccharide, was approved by the FDA for medical applications with consistent pharmaceutical parameters [46]. Taken together, these so-called VLMWHs or ultra-low-molecular-weight heparins (ULMWHs), including fondaparinux, showed a higher anti-Xa activity than other heparin macromolecules such as UFH or LMWHs. Therefore, these molecular-size-optimized ULMWHs or LMWHs have shown great potential in clinical applications as drugs with improved therapeutic effects and low toxic effects when compared with large UFHs [47]. The design of heparin derivatives can be optimized in terms of not only the molecular size and sequence but also the route of administration.

The oral delivery of heparin is important for patients with a high risk of clotting because the action of heparin is necessary for the prevention of thrombosis [22,48]. First, in order to improve the oral bioavailability of heparin, an oral formulation with an enhancer including sodium N-[8-(2-hydroxybenzoyl) amino] caprylate (SNAC) or sodium N-[10-(2-hydroxybenzoyl)amino] decanoate (SNAD) was developed [49,50,51,52]. Then, chemical heparin conjugates such as low-molecular-weight heparin and deoxycholic acid conjugate (LMWH–DOCA) or heparin–lipid conjugate were synthesized for oral heparin delivery [53,54,55,56]. These oral chemical conjugates have also been widely studied for the treatment of various other diseases such as cancer [13,57,58]. The problem with these synthesized heparin derivatives and conjugates is that at least five intact heparin saccharide rings are required for therapeutic action, but they can be easily lost during synthesis, increasing the complexity. Therefore, a precise end-site-specific conjugation of heparin to preserve its heparin sequence was attempted; then, a new enoxaparin and tetraDOCA conjugate (EnoxaTD) was developed using end-site-specific chemical glycosylation [59].

### 3.2. Heparin Conjugates for Anti-Cancer Therapy

The study of the use of heparins or heparin derivatives in cancer treatment began recently, showing promising results in terms of their ability to treat several tumors [60]. Cancer is one of the leading causes of death worldwide, and some cancer patients receive UFH or LMWH to prevent cancer-associated thrombosis (CAT) or blood coagulation. The use of heparin in cancer patients has shown various therapeutic effects; thus, scientists have attempted to modify heparin’s molecular structure to enhance its anti-cancer effects. In particular, heparin conjugates that have been chemically modified with other drugs or molecules have several advantages in terms of decreasing the bleeding toxicity of heparin as well as enhancing its biological activities. For example, when heparin binds to hydrophobic molecules such as cholesterol or bile acids, its anti-cancer effect can be increased along with a loss of its anticoagulant effect [61,62]. Various heparin and bile acid conjugates with taurocholic acid (TCA) or deoxycholic acid (DOCA) have been prepared and their ability proven, resulting in delayed tumor growth and metastasis [63,64]. Heparin–taurocholate conjugate (HT10) or heparin–DOCA conjugate (H-DOCA) were shown to inhibit tumor growth and metastasis while regulating the activity of tumor-related growth factors such as vascular endothelial growth factor A (VEGF A). VEGF A has a heparin-binding domain in its structure [65,66]. Recently, the target range of heparin derivatives was extended to tumor-related proteins such as transforming growth factor-β1 (TGF-β1), CXCL12, vascular endothelial growth factor C (VEGF C), and heparanase [67,68,69].

**Figure 3:**
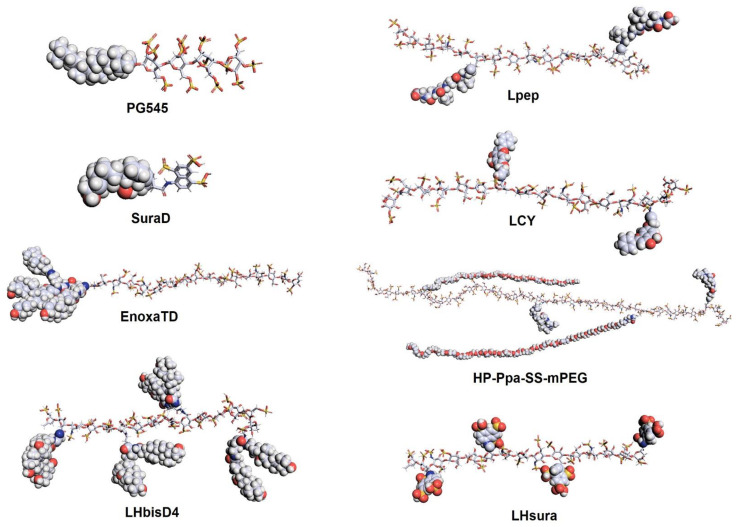
Chemical structures of recently studied heparin conjugates and heparin-mimic biomolecules. Heparin or the heparin-mimic moiety in the molecule is expressed as a stick, while the conjugate is expressed as a sphere. The molecular structures were visualized using the PyMOL (the PyMOL Molecular Graphics System, Version 2.5.0, Schrödinger) program. SuraD, suramin fragment and deoxycholic acid conjugate; EnoxaTD, enoxaparin and TetraDOCA conjugate; LHbisD4, LMWH and bisDOCA conjugate; Lpep, LMWH and peptide conjugate; LCY, LMWH and chrysin conjugate; HP-Ppa-SS-mPEG, PEG-detachable pyropheophorbide-a (Ppa)-functionalized heparin; LHsura, LMWH, and suramin fragment conjugate.

Heparin derivatives developed for anti-cancer treatment are not necessarily limited to heparin–steroid structures or derived from UFH. For example, PG545 is a heparin-like synthetic molecule with a hydrophobic cholestanyl aglycone moiety (Figure 3). It can show a strong anti-cancer effect, inhibiting angiogenic factors, with mild anticoagulant activity and stimulating immune responses against tumors [70,71,72]. Interestingly, it was recently reported that PG545 also displays potential for use as an anti-viral agent against SARS-CoV-2 and as a heparanase inhibitor with anti-lymphoma effects [73,74]. On the other hand, various function molecules such as tocopherol (for pH-triggered polymeric micelles) [75], biotin (for anti-heparanase activity to treat multiple myeloma) [76], chlorambucil (as a redox-responsive prodrug) [77], suramin fragment (to enhance or mimic heparin’s properties) [78,79], and the thiol group (for pH and GSH dual-responsive carriers for inhibiting tumor growth) [80] were recently conjugated to heparin molecules as new therapeutic biomolecules; they are currently under evaluation. In addition, heparin can be chemically modified with a hydrophobic photosensitizer (pyropheophorbide-a) for photodynamic therapy [81]. In a study, a polyethylene-glycol-modified (PEGylated) heparin and PDT conjugate achieved high tumor accumulation and had strong tumor-inhibitory effects. There was a complex study concerning the synthesis of a heparin derivative with matrix metalloproteinase (MMP2)-specific peptides for M2-to-M1-like macrophage reprogramming [82]. In the case of the heparin–peptide conjugate, heparin served not only as a hydrophilic biomaterial but also as a vessel for normalizing biomolecules contributing to anti-angiogenic effects. The results of studies conducted in animals showed that the heparin conjugate promoted potent tumor inhibition, anti-metastatic effects, and overall tumor microenvironment (TME) improvements. Taken together, heparin mimics, derivatives, and conjugates may be used in the design of efficacious and biocompatible therapeutics rather than unmodified heparins, as shown in Table 1.

### 3.3. Heparins as Anti-Viral Agents

Various anti-viral effects of heparin-based biomolecules were demonstrated in a recent study. In the case of influenza, a common flu virus, it is reported that heparin and heparin derivatives can have preventative effects through inhibiting the ability of the H5N1 strain of the virus to attach to cells [83]. This means that heparin derivatives and conjugates may be a potential source of viral inhibitors. In terms of recent world events, coronavirus disease 2019 (COVID-19) grabbed the attention of researchers focusing on advanced medication systems. Heparin treatment for COVID-19 patients has therapeutic potential; however, the exact role of heparin needs to be proven [104]. Some research has shown, in vitro, that heparin has promising anti-viral activity for the inhibition of SARS-CoV-2 (severe acute respiratory syndrome-related coronavirus-2) [105]. Another report has suggested that the administration of heparin to COVID-19 patients might be associated with lower mortality [87]. The use of heparins in COVID-19 patients may be safe; however, further clinical studies are needed to prove their therapeutic effect [106]. Regarding the use of heparins as anti-viral agents, it seems that UFH has stronger antiviral activity than LMWHs, inhibiting spike proteins in SARS-CoV-2 [107]. In addition, new anti-viral effects of heparin derivatives such as PG545, which were initially developed as anti-cancer drugs, have also been reported [74]. On the other hand, in response to the COVID-19 pandemic, the US Food and Drug Administration (FDA) recently decided to allow the use of a medical device named Seraph R100 that has heparin molecules on its surface for reducing bloodstream pathogens [108]. The surface of the heparin is designed to bond with the existing virus and remove it from the blood. These studies indirectly show that heparin may have value as a probe as well as a therapeutic agent. The development of heparin-utilizing devices or heparin derivatives for anti-viral effects might continue based on their protein binding and anti-viral ability.

## 4. Recent Nanoformulation of Heparin

**Figure 4:**
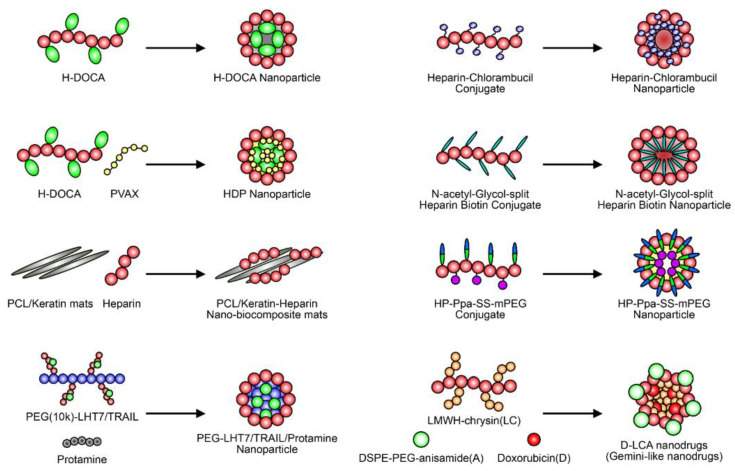
The schematic structures of nanoparticles based on heparin derivatives. Different types of heparin conjugates are currently being investigated as nanodrug carriers or new drug candidates. The self-assembled heparin–drug conjugate that forms after nanoformulation has shown great therapeutic potential for use in various therapies.

The use of nanoformulations of heparin or heparin derivatives for the treatment of various diseases has been shown to have several advantages [109]. Nanoformulations of drugs usually have several advantages, such as targeting effects, increased circulation times, and higher efficiency [110,111,112]. In the case of heparin, which is a highly hydrophilic biomolecule, the formation of nanoparticles is mainly achieved though the binding of hydrophobic organic molecules (Figure 4) [113]. For example, most heparin conjugates, including heparin–DOCA conjugates and bisdeoxycholyl–heparin (LHbisD4), which was developed to enhance oral absorption, can generate nanoparticles via self-assembly [114,115]. The redox-responsive heparin–chlorambucil conjugate that was recently developed by Andrgie et al., the heparin–biotin conjugates developed by Esposito et al., and the heparin-pyropheophorbide conjugate developed by Wu et al. can also self-assemble into spherical nanoparticles in an aqueous solvent [76,77]. Heparin conjugates are sometimes mixed with other polymers such as their antidote (protamine sulfate) [116,117] or anti-inflammatory polymer (copolyoxalate containing vanillyl alcohol, PVAX) [92] via co-assembly to increase the function and retention time of heparin in the blood, thus reducing its side effects on hemostasis. Heparin based on functional nanomaterials can be utilized for the treatment of various diseases. For example, Wan et al. recently conjugated poly(ε-caprolactone) (PCL) and keratin with heparin to bind VEGF [118]. Heparin/VEGF engineered materials show good biocompatibility upon testing for blood clotting time, hemolysis, and platelet adhesion. Taken together, it can be observed that there are many heparin-based nanoparticles that bear interesting characteristics for use in medical treatment. Considering the development of nanotechnology and heparin-based biomolecules, nanoformulations of heparin should be further studied in the future.

## 5. Conclusions and Perspectives

Heparin and heparin derivatives have shown great therapeutic potential as anticoagulants or functional biomolecules in many polysaccharide studies. However, the clinical use of heparin for the treatment of thromboembolism or atrial fibrillation has been challenged with the advent of DOACs such as rivaroxaban and apixaban. Although heparins including UFH and LMWHs are highly safe and effective, their use in the clinic is limited to parenteral administration due to their poor oral bioavailability. Considering the importance of anticoagulants for use in prophylaxis, it is highly advantageous that DOACs can be taken orally every day [33,119]. Therefore, although the increasing use of DOACs is inevitable, both heparins and DOACs will likely be widely used clinically because of the unique safety profile and effectiveness of heparin [120,121,122,123,124]. On the other hand, research on the non-coagulant effects of heparin based on their therapeutic potential in various clinical situations is ongoing. Due to the other functional properties of UFH and LMWH observed, they have been utilized for applications besides being used as anticoagulants [125]. Many preclinical studies and experimental research papers have shown that heparin can be considered a modulator of growth factors, cell-adhesion molecules, chemokines, viral proteins, immune factors, and endothelial cells [42,126,127,128]. Even without the anticoagulant effect of heparin, it is clear that its number of new applications seems set to increase in the near future.

In this review, we propose that chemical modification or conjugation with heparin molecules can improve the use of heparin as a therapeutic in various diseases. These approaches might be able to greatly expand the current treatment options for heparins in clinical settings with high efficacy and low toxicity, overcoming the current limitations of their clinical application as anticoagulants. In this regard, several studies have evaluated and clinically analyzed the therapeutic functions of various heparin-based biomolecules. Recent advances in the preparation of heparin-related biomolecules or nanoformulations of heparin conjugates include the emergence of heparin derivatives or conjugates that are suitable for successful clinical applications. In this article, we summarized the recent advances in the development of heparin derivatives and conjugates as potential therapeutics. Considering the current limitations of the use of heparin, we expect that new heparin derivatives and conjugates will provide us with opportunities for further clinical applications.

## Figures and Tables

**Table 1:** Recently developed heparin-based biomolecules for the treatment of various diseases.

Class	Molecular Effects	Materials	Highlight	Year	Refs.
Heparin	Inhibits influenza H5N1	Chemically modified heparin	A H5 pseudotyped HIV system	2015	[83]
Heparin	Chronic kidney disease	Tinzaparin	CrCl ≥ 20 mL/min in patients	2019	[84]
Heparin	Transduction efficiency	Enoxaparin	pLV-S ^1^ typed virus	2021	[85]
Heparin	Sepsis inhibition	LMWH	Patients with COVID-19	2020	[86]
Heparin	Coagulopathy in COVID-19	LMWH	Change in survival rates	2020	[87]
Heparin	Wound healing	Heparin	Hemostatic protein, VWF ^2^ deficiency	2019	[30]
Synthetic molecule	Heparanase inhibitor	Pixatimod	Directly binds to S1 protein RBD of SARS-CoV-2	2020	[88]
Synthetic molecule	Heparanase inhibitor	Roneparstat	Myeloma therapy	2018	[89]
Nanocarrier	Hybrid nano-thin film	Heparin/peptide–polyethylene glycol	Store morphogen	2018	[90]
Nanocarrier	Anti-cancer activity	AIB1 ^3^ siRNA-loaded PEI/heparin/Ca^2+^ NPs	A non-viral polymer carrier for AIB1 siRNA	2018	[91]
Nanocarrier	Anti-thrombotic	Hp-DOCA-PVAX ^4^ nanocomposite	Reduced inflammation and coagulation	2019	[92]
Nanocarrier	Malaria therapy	Artesunate–heparin conjugate-based nano-capsules	*P. falciparum* inhibition	2019	[93]
Nanocarrier	Photodynamic therapy	Hp-Ppa-SS-mPEG ^5^	Increased ROS production and apoptosis	2021	[81]
Nanocarrier	Human colon adenocarcinoma	Chitosan/heparin polyelectrolyte complexes	Oral hydrophilic drugs	2021	[94]
Nanocarrier	Anti-tumor and anti-angiogenic efficacy	Dalteparin-Poloxamer with LR-DOX ^6^ hydrogel	Exhibiting a good thermosensitivity	2019	[95]
Conjugate	Heparanase	With biotin	Metastasis inhibition	2020	[96]
Conjugate	Anti-tumor and anti-angiogenic efficacy	PEG-LHT7/TRAIL/protamine nanocomplex	Increased tumor-resident time for TRAIL	2021	[97]
Conjugate	Anti-angiogenic activity	Suramin fragment–DOCA	Binding with HBD ^7^ of VEGF ^8^	2021	[78]
Conjugate	GAG ^9^-based COVID-19 therapeutics	Heparin–amine–PEG_3_–biotin	SARS-CoV-2 ^10^ glycoprotein binding	2020	[98]
Conjugate	Anti-cancer activity	Heparin–chlorambucil	High redox potential.	2019	[77]
Conjugate	Anti-cancer activity	Heparin–α-tocopherol–docetaxel	Increased cytotoxicity against cancer cells	2020	[75]
Conjugate	Protein interactions	Biotin–heparin	At low temperature	2018	[99]
Conjugate	Anti-cancer activity	Heparin–SH–doxorubicin	High biocompatibility	2020	[80]
Conjugate	Improved anti-angiogenic activity	ES2-GSHP ^11^	Wide pH activity range and a longer half-life	2019	[100]
Conjugate	Anti-cancer activity	PCLA–PEG–PCLA ^12^ polymeric hydrogel	Temperature-responsive hydrogel	2019	[101]
Conjugate	Anti-corneal neovascularization	LMWH	Distribution level needs every 4 to 6 h	2018	[102]
Conjugate	Anti-microbial activity	Piscidin–heparin	Cu^2+^ interaction	2018	[103]

^1^ pLV = lentiviral vector; ^2^ VWF = von Willebrand factor; ^3^ AIB1 = nuclear receptor coactivator 3; ^4^ Hp–DOCA–PVAX = heparin–deoxycholic acid copolyoxalate containing vanillyl alcohol; ^5^ Hp–Ppa–SS–mPEG = Hp-based polymer conjugate with pyropheophorbide; ^6^ LR–DOX = laponite RDS–doxorubicin; ^7^ HBD = heparin-binding domain; ^8^ VEGF = vascular endothelial growth factor; ^9^ GAG = glycosaminoglycan; ^10^ SARS-CoV-2 = severe acute respiratory syndrome-related coronavirus 2; ^11^ ES2–GSHP = endostatin2-glycol-split heparin; ^12^ PCLA–PEG–PCLA = (poly-(ε-caprolactone-*co*-lactide)–*b*-poly (ethylene glycol)–*b*-poly(ε-caprolactone-*co*-lactide).

## Data Availability

Not applicable.
